# A cautionary tale about using the apparent carbon accumulation rate (aCAR) obtained from peat cores

**DOI:** 10.1038/s41598-021-88766-8

**Published:** 2021-05-05

**Authors:** Dylan M. Young, Andy J. Baird, Angela V. Gallego-Sala, Julie Loisel

**Affiliations:** 1 School of Geography, University of Leeds, Leeds, UK; 2 Geography Department, College of Life and Environmental Sciences, University of Exeter, Exeter, UK; 3 Department of Geography, Texas A&M University, College Station, TX 77843 USA

**Keywords:** Ecological modelling, Wetlands ecology, Hydrology

## Abstract

The carbon (C) accumulation histories of peatlands are of great interest to scientists, land users and policy makers. Because peatlands contain more than 500 billion tonnes of C, an understanding of the fate of this dynamic store, when subjected to the pressures of land use or climate change, is an important part of climate-change mitigation strategies. Information from peat cores is often used to recreate a peatland’s C accumulation history from recent decades to past millennia, so that comparisons between past and current rates can be made. However, these present day observations of peatlands’ past C accumulation rates (known as the apparent rate of C accumulation - aCAR) are usually different from the actual uptake or loss of C that occurred at the time (the true C balance). Here we use a simple peatland model and a more detailed ecosystem model to illustrate why aCAR should not be used to compare past and current C accumulation rates. Instead, we propose that data from peat cores are used with existing or new C balance models to produce reliable estimates of how peatland C function has changed over time.

## Introduction

Containing over 500 billion tonnes (Pg) of carbon (C)^1–3^, peatlands are an important part of the global C cycle, and there is considerable interest in how C accumulation in these systems has varied in the past and how it might respond to future changes in climate and land management^4–8^. Carbon accumulates in a peatland because more plant material is added to it than is lost via decay. Although rapid in the near surface (often called the acrotelm), decay rates in waterlogged deeper peat (the catotelm) are much lower, allowing peat to build up. However, the reverse can happen at times; more material can be lost than is added, resulting in a decrease in a peatland’s C store and a net release of C to the atmosphere (e.g.^6,9^).

To reconstruct the C accumulation history of a peatland^10^, scientists calculate what is often called ‘aCAR’^11^: the apparent Carbon Accumulation Rate. To estimate aCAR, it is necessary first to establish the age of the peat down a peat core; this is done by dating samples of peat from a number of depths and fitting a curve through the data^12,13^. The C content of contiguous or regularly-spaced layers of peat down the core also needs to be measured. aCAR is then the amount of C (per unit area) in a layer divided by the difference in age between the top and bottom of the layer. aCAR is usually plotted against time to infer how rates of C accumulation have varied during a peatland’s developmental history. Although aCAR is a widely-used metric, problems with its interpretation have been discussed over many years (e.g.^10,11,14–19^).

aCAR has a number of problems as an indicator of the net C accumulation rate of a peatland. First, because it is a measure of the amount of C found within a peat layer *at the time of coring* it is dependent on the overall age of the layer; this is because decay of the layer will continue as the layer gets older—the layer loses mass and C over time since its formation. For example, the aCAR for peat that is 3,000 years old may be greater than for peat in the same profile that is 4,000 years old. However, this difference in aCAR does not mean the peatland was necessarily accumulating C more rapidly 3,000 years ago than it was 4,000 years ago: when the layer of 4,000-year old peat was only 3,000 years old, its aCAR may have been the same as that of the layer of peat that is currently 3,000 years old. This ‘ageing’ problem makes it impossible to use aCAR to determine the true net rate of C accumulation of a peatland over time. A second problem is that aCAR does not account for what else may be happening to other layers in the peat profile^10,11^. When a new peat layer is being formed at the peatland surface, new C is being added to the profile, but a greater amount of older C may be being lost via decay from the rest of the peat profile below the new layer. Therefore, despite new C being added, the peatland as a whole may be losing C. This net loss of C is not part of the calculation of aCAR, which is a measure solely of how much C has been added to a peatland over a period of time (notwithstanding ongoing losses from the layer because of decay). Therefore, aCAR can only ever give positive values of C accumulation within the time period spanning the layer of interest^11^.

Further problems in the interpretation of aCAR arise when it is calculated for near-surface peat (i.e., peat from recent decades), and these problems have been termed the ‘acrotelm effect’. We recently explained^10^ why aCAR from near-surface peat cannot be reliably compared to the long-term rate of C accumulation obtained from a deeper layer within a peat core, which may be several centuries, or more, old. Greater values of aCAR found in the near surface of a peatland are an artefact that arises because recently-added plant litter has decomposed much less than older, deeper, peat. The artefact is another example of the peat-ageing problem noted above, but it is exacerbated in the acrotelm because of the higher decay rates in this aerated part of the peat profile. As a result, many peatland scientists choose to ignore aCAR in the upper parts of the peat profile; they are aware of the problem and do not use or attempt to interpret the increase in aCAR in progressively younger peat (e.g.^20^). However, misinterpretation of the effect is common in the recent literature (e.g.^21–24^). These studies also mistakenly assume that aCAR, when calculated for the acrotelm as a whole (by treating the acrotelm as a single layer), gives a multi-decadal average net rate of peat C accumulation for the entire peat profile, when in fact it merely describes the C content of the acrotelm. This erroneous assumption is perhaps most prominently made by Rydin and Jeglum^25^, who suggest that:

“… it may be useful to have a measure of peat accumulation over the last few decades …This measure is the recent rate of carbon accumulation (RERCA), which is obtained from the bulk density [which gives the mass of peat and C] down to a dated level not far from the surface. Given the recent developments in precise and accurate dating of young peat…, this is now quite possible” [text in brackets added].

As defined by Rydin and Jeglum^25^ RERCA is simply aCAR calculated for a single layer of peat at the peatland surface, and, as noted above, this layer may comprise all of the acrotelm. Rydin and Jeglum^25^ note that RERCA may be used instead of direct measurement of the C fluxes to and from a peatland (see below); in doing so, it is clear they assume RERCA is a measure of the C budget of the peatland in its entirety.

Data from peat cores are, of course, essential for understanding peatlands’ C accumulation histories, but it is their use to calculate aCAR or RERCA that we wish to challenge. In discussions with peatland scientists and policy makers we have realised that the problems with aCAR and RERCA are still not fully appreciated; in particular there is confusion over why RERCA cannot be used to give an average net C accumulation rate for a peatland as a whole over recent decades (e.g.^26^). To address these misunderstandings, and to expand on the explanations given in^10^, we present and discuss here the results from a simple numerical model based on Clymo’s^14^ work and a more detailed computer model of peatland development. The simple peatland model is used to illustrate, from first principles, how the acrotelm effect arises. We show how an increase in aCAR in the uppermost part of the peat profile arises even when: (1) actual rates of net C accumulation for the peatland as a whole decline over time, (2) net C accumulation rates for the peatland are steady (constant) over time, and (3) net C accumulation rates are negative (there is a net loss of C from the peatland as a whole). We also show why, except in one unusual case, RERCA is not equal to the net rate of C accumulation of the peatland. Specifically, we show how the method used to calculate RERCA is based on a misapplication of the mass balance equation. In the second part of the paper, we use the DigiBog peatland development model^10,27^ to show how the mismatch between aCAR and actual rates of net C accumulation, explained by our simple peatland model, applies over millennial timescales. DigiBog’s outputs enable us to calculate aCAR and actual (‘true’) rates of net C accumulation for the thousands of years over which our simulated peatlands develop. Because climate over such timescales is rarely constant, we also explore the effect of changes to temperature and net rainfall (precipitation minus evapotranspiration) on net C accumulation and aCAR.

In the remainder of the paper we use a third acronym to describe the rate of C accumulation in peatlands: NCB or net carbon balance^11,28^. aCAR and RERCA (which, as noted above, is a special case of aCAR) are both calculated for layers of peat. NCB, in contrast, includes all C additions to, and all C losses from, a peatland and may be thought of as the true rate of net C accumulation for the whole peatland (or the whole peat column) at a particular time. NCB may be obtained directly by measuring atmosphere-peatland C exchanges using flux towers and by measuring C losses in water discharging from a peatland (e.g.^29,30^).

### Conceptualising the acrotelm effect

**Figure 1:**
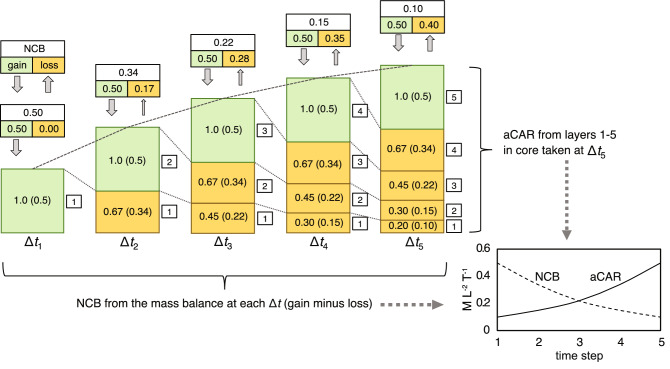
Scenario 1 showing the establishment phase of a peatland. Changes to a single column of peat of unit area are shown for five separate timesteps (Δ*t*). Newly-added litter/peat is shown in pale green. Older peat is shown in pale brown/orange. The numbers in each layer of peat show the mass of peat and, in brackets, the mass of carbon, both per unit area (arbitrary units). NCB denotes the actual or real rate of net C accumulation and is given by the gains of C (in new litter) minus the loss of C from the decay of the older peat cohorts for each Δ*t*, as shown in the boxes above the columns. The arrows below the boxes represent gains (down-pointing) and losses (up-pointing) of C. aCAR is calculated for each cohort or layer at Δ*t*_5_. The graph shows NCB and aCAR for each Δ*t*. In Scenario 1, the water table always resides at the base of the peat column; there is no catotelm, and all of the peat is aerated—it is acrotelm peat.

To illustrate how the acrotelm effect arises we first use a very simple numerical model where litter produced by peatland plants is added to the peatland surface as cohorts or layers. The rate of litter production has dimensions of mass (addition) per unit area of peatland per time (M L^−2^ T^−1^). Past cohorts (M L^−2^) decay at a specified proportionate rate (proportion per time—T^−1^) in accordance with Clymo^14^. We consider three scenarios. In Scenario 1, which we term the ‘establishment phase’ of a peatland, new peat forms on, for example, a bare mineral surface. This phase is illustrated for a column of peat in Fig. 1. In the model shown in the figure, the peatland grows over a period of five notional timesteps (Δ*t*_1_–Δ*t*_5_). Throughout this period, litter production is constant at a rate of 1.0 per timestep (arbitrary mass units per unit area), while decay occurs at a fixed proportionate rate of 0.33 per timestep*.* The peat column is shown for each timestep and its height is proportional to the total mass of peat (M L^−2^) (Fig. 1). During Δ*t*_1_, 1.0 mass unit of litter (per unit area) is added and no peat is lost to decay. Therefore, the net mass accumulation rate is 1.0. During Δ*t*_2_ 1.0 mass unit of new litter is again added, but 0.33 mass units of old litter or peat are lost from the pre-existing cohort (formed during Δ*t*_1_), so that its mass is reduced to 0.67. Therefore, by the end of Δ*t*_2_ the peatland contains 1.67 mass units compared to 1.0 at the end of Δ*t*_1_. In other words, the net gain or accumulation rate has reduced to 0.67 in Δ*t*_2_ from 1.0 in Δ*t*_1_. This pattern continues and the peatland continues to grow, but at a decreasing rate, during the remaining timesteps (Δ*t*_3_–Δ*t*_5_) as shown in the figure. The actual C balance (NCB) of the peatland is also shown in the figure, where it is assumed that C comprises half of the mass of the litter/peat (see below).

A scientist wishing to know rates of net peat and C accumulation (NCB), and how these change over time, during the establishment phase (i.e., between Δ*t*_1_ and Δ*t*_5_), might measure, at each time step, C fluxes to and from the peatland directly (see "Introduction" and reference to^29,30^). Alternatively, they could core the whole peat profile at each of the time steps and measure the peatland’s *total* C content on each occasion and see how it changes over time. In practice, neither approach may be practicable because of the time span involved. Most research projects last a few years (typically three to five years), and even long-term monitoring programmes rarely exceed one or two decades. Therefore, if Δ*t*_1_–Δ*t*_5_ spanned more than a few decades, the period would be much too long for most studies. Because of these practical difficulties, an alternative used by some peatland scientists is to take a peat core from the peatland in its current state (Δ*t*_5_ in Fig. 1) and to use the core to reconstruct the apparent C accumulation history of the peatland. This reconstruction is done by dating the peat in the profile and by measuring the mass of layers of peat for which the ages of the upper and lower boundaries of the layers are known (i.e., the duration of the interval represented by the layer is known). For the simple case in Fig. 1, we may assume that the layers for which aCAR is calculated are coincident with the cohorts of litter added every timestep (Δ*t*).

If we assume that the proportion of the peat mass that is C is 50%^17^, NCB is simply half the value of the mass accumulation rate discussed above. Therefore, for Δ*t*_1_, 0.5 mass units of C are added and none are lost. For Δ*t*_2_, 0.5 mass units of C are again added, but 0.17 C units are lost from the existing layer formed during Δ*t*_1_ (loss being the mass in the layer times the decay rate: 0.5 × 0.33 = 0.17), giving a net rate of C accumulation of 0.34 (Fig. 1). As noted above, the peatland continues to gain mass and C but the rate of gain decreases with time. This decrease in rate of net peat and C accumulation is shown by the grey dashed line connecting the top of peat columns in Fig. 1 and is what would be indicated by direct measurement. In contrast, aCAR erroneously suggests that the rate of growth is *increasing* over time because it does not consider decay other than in the dated layer of interest. The uppermost layer of peat representing the last timestep (Δ*t*_5_) appears to accumulate at a greater rate than the older cohorts that have undergone progressively more decay with age. For example, the first or deepest cohort, formed in Δ*t*_1_ (layer 1 in the figure), has an initial C content of 0.5, which, through decay, becomes 0.34, 0.22, 0.15, and finally 0.1 by the end of Δ*t*_5_. Ascending from this deepest layer, aCAR increases to the surface, giving a pattern that is the opposite of the real rate (NCB), as shown by the graph in the lower right of Fig. 1.

Two fundamental differences between aCAR and NCB are revealed here. First, NCB is measured in ‘real time’ (the fluxes are estimated for the time period in which they occur), whereas aCAR is measured between dated layers in a peat core that may have been taken many decades or centuries after the layer was first formed (at the end of Δ*t*_*5*_ in our example). This ‘delay’ means that aCAR does not take account of changes to a cohort of peat after its initial formation. For example, if the peatland in Fig. 1 had been cored at the end of Δ*t*_4_, rather than at the end of Δ*t*_5_, the aCAR calculated for the lowermost cohort of peat in the profile (layer 1) would be 0.15 and not 0.1. Therefore, *aCAR is dependent on the time at which the peatland is cored*. This dependency is the ageing effect noted in the Introduction.

Secondly, and more importantly, unlike NCB, *aCAR does not consider what happens in the whole profile*, which is necessary when constructing a whole-peatland C budget. For example, NCB during Δ*t*_4_ comprises litter addition but also decay losses from the litter laid down in the previous three timesteps, giving a value of 0.15 (inputs of 0.5 minus decomposition losses of 0.35—see Fig. 1). When aCAR is calculated for the same time period—i.e., Δ*t*_4_—it considers only the remaining mass of new peat laid down during Δ*t*_4_, giving a value of 0.34 (for a core taken at the end of Δ*t*_5_).

If aCAR is calculated for the column of peat as a whole at Δ*t*_5_ to give RERCA (i.e., the C mass of the whole core, given by 0.5 + 0.34 + 0.22 + 0.15 + 0.10 = 1.31 units of C, divided by 5 [timesteps]), it will give the correct average NCB (0.26) for the period between Δ*t*_1_ to Δ*t*_5_. This correspondence in values may be regarded as unusual because the acrotelm comprises the whole peatland in Scenario 1; using the whole peat profile means that all additions and losses are accounted for, making it impossible for RERCA to give anything other than the right value. However, in most situations the acrotelm sits atop a catotelm and there won’t be a correspondence of values between RERCA and NCB, as we show below in the next two scenarios and also in the next section (‘RERCA and net C balance are not comparable in near surface peat: the peatland mass balance equation and its misuse’).

**Figure 2:**
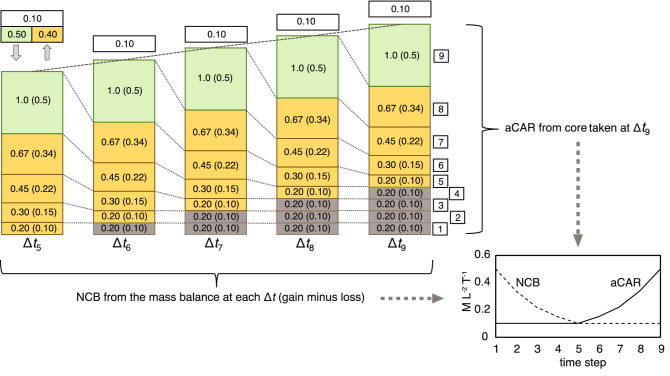
Scenario 2 showing a dynamic acrotelm of constant mass, and a steadily-thickening catotelm (blue-grey shading representing waterlogged peat). Layers 1–4 become submerged by the rising water table, and by Δ*t*_9_ the acrotelm comprises layers 5–9.

It may be argued that the situation in Fig. 1 is too simple because there is no lower zone of waterlogged peat; there is no catotelm, as seen in most peatlands. In Scenario 1, the water table resides at the bottom of the peat profile—all of the peat is in the acrotelm—and all litter/peat decay occurs at the same (oxic) proportionate rate. We can, however, extend the model by assuming that older, more decayed peat, is less permeable^14^ and that water drains less readily through it, causing the water table to rise. Figure 2 shows one realisation of this possibility (Scenario 2). In the figure, the acrotelm is in a dynamic equilibrium: its mass remains constant, but new litter continues to be added to it at a constant rate, while the oldest cohorts at the bottom become part of the waterlogged catotelm as the water table rises above them. In Scenario 2, the cohorts that have reached a specified degree of decay (80%, or cohorts with a remaining mass of 0.2) are transferred to, or become part of, the catotelm. Therefore, cohorts added to the top of the acrotelm are buried under new litter and continue to decay until they become part of the catotelm. This situation is similar to that modelled by Clymo^14^, with the main difference being that peat below the water table in Scenario 2 is assumed not to decay at all (Clymo^14^ allowed for a low rate of decay). The peatland accumulates mass at a constant rate, as shown by the straight dashed lines fitted to the top of the peat profile in Fig. 2.

What happens if we core the peatland in Scenario 2 at the end of Δ*t*_9_ and calculate aCAR for each of layers 1–9? We see that aCAR for layers 1–5 has now changed to 0.1 compared to the ascending values obtained when the peat was cored at Δ*t*_5_ (Fig. 1). This difference is because these layers have now decomposed further before becoming part of the catotelm when decay ceased. An apparent increase in rates of C accumulation is still evident, however, but now in the layers of peat formed between Δ*t*_5_ and Δ*t*_9_ (layers 5–9) that lie above the water table and form the acrotelm.

Both aCAR and NCB are plotted against time in the graph in the lower right of Fig. 2. aCAR shows a pattern similar to that from many real peat cores: a low and relatively stable aCAR in the older parts of the peat core, with an increase as one approaches the peatland surface^10^. It is instructive to compare these values with NCB. NCB was high initially when the peatland first formed (Scenario 1: Δ*t*_1_ to Δ*t*_5_) and declined to a steady value (Scenario 2: Δ*t*_6_ to Δ*t*_9_). As with Scenario 1, aCAR erroneously suggests that the net rate of C accumulation has increased to the present, and only one of the aCAR values corresponds to NCB (0.1), now for Δ*t*_5_ (layer 5). In contrast to Scenario 1, RERCA (again applied to the acrotelm as a whole) is now wrong, giving a value of 0.26 ((0.5 + 0.34 + 0.22 + 0.15 + 0.1)/5 [timesteps]), instead of the correct value of 0.1.

**Figure 3:**
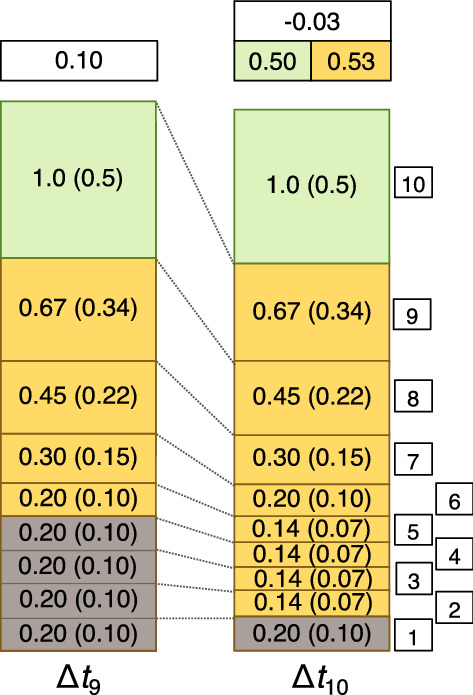
Scenario 3 showing a net loss of peat mass caused by the secondary decay of previously waterlogged layers of peat. During the drought in Δ*t*_10_ the water table falls to the top of layer 1, exposing previously ‘protected’ peat in layers 2–5 to oxic decay (secondary decay).

Finally, in Scenario 3 we may consider what happens if the peatland experiences a drought that causes the water table to fall so that layers that were in the catotelm and below the water table are now exposed above it and undergo renewed or ‘secondary’ oxic decay^9^. A realisation of this situation is shown in Fig. 3, where the peatland, overall, loses mass during Δ*t*_10_. During the timestep, the peatland gains 1.0 mass units of litter but loses 1.06 mass units via decay of existing layers of peat above the drought water table (the layers laid down during Δ*t*_2_–Δ*t*_9_), giving a net rate of accumulation of − 0.06. For C the figures are a gain of 0.5 and a loss of 0.53, giving a net loss of 0.03 mass units of C per unit area (Fig. 3). If the peatland is cored at the end of Δ*t*_10_ and aCAR calculated, the same problems as identified before are evident. aCAR suggests that C accumulation is increasing over time to the present. In addition, in this scenario not only does RERCA, when applied to the acrotelm as a whole, give the wrong value of net C accumulation, it also gives the wrong sign. In Scenario 3, RERCA suggests a net C accumulation rate of 0.18 (when calculated for the now deeper acrotelm incorporating the cohorts formed between Δ*t*_2_ and Δ*t*_10_), when in fact the peatland as a whole has become a net source of C. Here, we repeat an important point made by^11^: *aCAR cannot be negative*.

Other scenarios in addition to the three discussed here are possible, such as ones that include changes in rates of litter production as well as changes in decay in response to drought (a modification of Scenario 3), and these may even lead to a decrease in aCAR towards the top of a core. However, the three scenarios have, between them, sufficient generality for revealing why aCAR is an unsatisfactory measure of C accumulation in peatlands. All peatlands are expressions of the balance equation: organic matter is added via litter production and is lost via decay (and sometimes erosion—not considered here). Therefore, regardless of differences in specific production and decay rates, scenarios akin to those considered above may arise in all types of peatland. In other words, the problems identified with the use of aCAR in each scenario apply regardless of the values of litter production and the decay coefficient that are used. It is clear that aCAR is misnamed; it is *not* a measure of net C accumulation rate—it never can be because of the way it is calculated. In the next section we extend our analysis to show that the calculation of aCAR is based on an erroneous version of the peatland mass balance equation. For simplicity, we confine our analysis to RERCA, which, as we note severally above, is aCAR applied to the uppermost part of the peat profile spanning the most recent decades in a peatland’s history; often, the acrotelm as a whole.

### RERCA and NCB are not comparable in near surface peat: the peatland mass balance equation and its misuse

An advantage of the simple peatland model is that the problems associated with calculating aCAR for near-surface peat become readily apparent. While it is not difficult to grasp intuitively how the artefact of an apparent increase in rates of net C accumulation arises, the exact cause of the apparent increase can be easily identified when cohorts of litter or peat are tracked over time as in Scenarios 1 and 2 (Figs. 1, 2). The simple model is, however, even more useful when considering the problem of RERCA. Without recourse to the simple model, it seems reasonable to suggest that the mass of the acrotelm divided by its overall age (obtained by dating the peat at its base), gives a reliable ‘bulk’, or time-averaged, estimate of the net rate of C accumulation for the peatland as a whole. In Fig. 1 we show that this suggestion is correct for a newly-formed acrotelm (because, in this case, all additions and all losses are considered), and it is tempting to think that it also applies to other situations. After all, the acrotelm contains new peat added in the years since the date of the acrotelm-catotelm boundary, so this would appear to be a net gain to the peatland, especially if there is little or no decay in the underlying catotelm.

Our simple peatland model shows why this apparently reasonable view is mistaken in more typical situations (more typical than Scenario 1) where the acrotelm is already in existence and does not develop from scratch, and where a catotelm is present. Scenario 2 is one such situation. Here, the extant acrotelm has a fixed mass, but is dynamic: mass is added to it via litter production and mass is lost from it via decay and transfer to the catotelm (the latter caused by water-table rise). Conceptually, the acrotelm can be thought of as a simple store. To obtain an estimate of the rate of net mass addition or loss, it is necessary to look at the *change* in the store’s mass over time. In equation form, where *I*_*a*_ is litter input rate to the acrotelm (M L^−2^ T^−1^), *O*_*a*_ is output rate from the acrotelm (decay as well as transfer of peat to the catotelm) (M L^−2^ T^−1^), *S*_*a*_ is the amount of mass in the acrotelm store (M L^−2^), *t* is time (T), and *i* is time level, we can write the balance equation thus:

1 $${I}_{a}-{O}_{a}=\Delta {S}_{a}/\Delta t=\left({S}_{a,i}-{S}_{a, i-1}\right)/\left({t}_{i}-{t}_{i-1}\right)$$

What this equation shows is that, if we measure Δ*S*_*a*_/Δ*t*, we can obtain the rate of net mass addition (*I*_*a*_ − *O*_*a*_) in the acrotelm. In Scenario 2 (Fig. 2), we see that Δ*S*_*a*_ between any of the time steps is zero, meaning that *I*_*a*_ − *O*_*a*_ is also zero; there is no net accumulation of peat or C in the acrotelm. For example, at the end of Δ*t*_7_ *S*_*a,*7_ is 2.62 (1.0 + 0.67 + 0.45 + 0.30 + 0.20) (for C, the figure is half of this). At the end of Δ*t*_8_ *S*_*a,*8_ has the same value (although some different cohorts are now involved because the acrotelm has migrated upwards). Therefore, the right hand side of Eq. (1) gives (2.62–2.62)/(8–7) = 0. Δ*S*_*a*_/Δ*t* is zero, as is *I*_*a*_ − *O*_*a*_.

Equation 1 may be rendered *wrongly* as follows:

$${I}_{a}-{O}_{a}={S}_{a}/\Delta t={S}_{a, i}/\left({t}_{i}-{t}_{i-1}\right)$$

Δ*S*_*a*_/Δ*t* has been replaced by *S*_*a*_/Δ*t*. Here, net peat and C accumulation is being estimated from the mass in the acrotelm *at one time only*. This erroneous version of Eq. (1) is what is used when calculating RERCA, where *S*_*a*_,_*i*_ is the current mass of the acrotelm (i.e., at *t*_*i*_) and *t*_*i*-1_ now represents the age at the base of the acrotelm. If we apply this version of Eq. (1) to Scenario 2, we obtain 2.62 [the mass of peat per unit area held in the acrotelm]/5 [the difference in age between the peat at the top and bottom of the acrotelm] = 0.524 mass units per unit area per timestep, instead of the correct Δ*S*_*a*_/Δ*t* value of zero. In C terms, the value is 0.262 C units per unit area per timestep (again, instead of the correct value of zero). This erroneous version of the equation can generally only produce the right result in the specific and unusual case where the mass in the acrotelm at *t*_*i*−1_ (*S*_*a,t*−1_) is 0 (i.e., Scenario 1).

However, there is a further problem here; the change in mass of the catotelm has been ignored. As noted above, the acrotelm loss term (*O*_*a*_) includes two components: the loss of peat to decay and the transfer of peat from the acrotelm to the catotelm. Only the former represents a loss from the peatland; the latter remains part of the peatland and should not, therefore, be included in the loss term when calculating the net C balance of the peatland. In other words, it is not enough to look at the acrotelm alone when estimating the C budget of the peatland as a whole, *even when decay in the catotelm is zero*. When estimating the net rate of C accumulation for the whole peatland, a balance equation that includes both the acrotelm and the catotelm is needed:

2 $${I}_{a}+{I}_{c}-{O}_{a}-{O}_{c}=\left(\Delta {S}_{a}+{\Delta S}_{c}\right)/\Delta t=\left({S}_{a,i}-{S}_{a, i-1}+{S}_{c,i}-{S}_{c,i-1}\right)/\left({t}_{i}-{t}_{i-1}\right)$$

where the subscript *c* denotes the catotelm.

Calculated correctly, the net C balance of the peatland in Scenario 2 between *t*_7_ and *t*_8_, for example (see above), is therefore (1.31 − 1.31 + 0.3 − 0.2)/(8 − 7) = 0.1 as shown in Fig. 2.

In Scenario 2 we could have allowed the catotelm to decay slowly at an anoxic rate, which would have meant that the rate of peat accumulation would decrease very slightly over time, but this would not alter our main finding that aCAR wrongly suggests a rapid increase in rates of accumulation. In fact, the discrepancy between aCAR and NCB would be even larger in such a situation from *t*_5_ onwards; therefore, our assumption is conservative. What this simple analysis shows is that measurements in the acrotelm alone cannot, except in special cases, be used to provide information on the overall C balance of a peatland. In other words, RERCA is based on a misuse of the balance equation: *to estimate the mass balance of the peatland as a whole, it is necessary to measure all of its components*.

Equation (2) allows for situations where the catotelm gains mass and C and where it is a net loser. However, it can sometimes be unclear where the boundary of the acrotelm and catotelm should be drawn. For example, in Scenario 3, should the acrotelm include layers 2–5 or not? It may be preferable to think of ‘acrotelm’ and ‘catotelm’ as somewhat contrived entities^31^, in which case the peatland should be considered a single store, giving:

3 $${I}_{p}-{O}_{p}={\Delta S}_{p}/\Delta t=\left({S}_{p,i}-{S}_{p, i-1}\right)/\left({t}_{i}-{t}_{i-1}\right)$$

where the subscript *p* denotes ‘peatland’.

Our simple peatland model and mass balance equations demonstrate why aCAR, and the special case of RERCA (aCAR for recent peat accumulation), cannot be used to understand changes to peatland C accumulation. However, peatlands develop over millennia and include a wide range of processes including feedbacks^32^ that can mediate their response to climate and land use, which are not represented in the simple model. We therefore used a more detailed process-based model (DigiBog) to simulate peatland development over thousands of years and to explore the dynamics of aCAR and NCB in response to perturbations to our model’s driving data.

### Simulating the effect on aCAR and NCB of changes in climate

We used the DigiBog peatland development model^10,27^ to ‘grow’ a sloping blanket peatland from the north of England over six millennia (see "Methods"). Our model simulates the peatland as a series of linked columns of peat. These can gain or lose mass (including C) depending on the climate inputs, simulated land uses and the autogenic mechanisms of the virtual peatland. And because the model records *during the simulation* the height of each peat column (based on the addition of mass to the peatland surface and the change in mass of each sub-surface peat layer), we can calculate the rate of change in the mass of C at each time step ($\Delta t$)—i.e. we know a peat column’s or the peatland’s NCB throughout the whole developmental history of the peatland (see "Methods"). At the end of a simulation we can also take a virtual core for a column and, as previously described, use the difference in age between the top and base of the layers within it, to calculate aCAR (see "Methods"). Because NCB must be calculated at the time the C fluxes occur, it is only possible to compare these past long-term dynamics of aCAR and NCB by using a peatland model.

**Figure 4:**
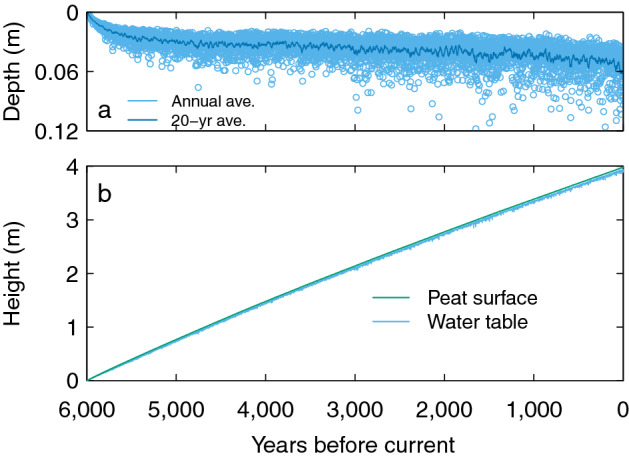
Development of the virtual blanket peatland over six millennia. (**a**) Water-table depth and (**b**) the peat surface from the virtual core at the centre of the peatland from the baseline simulation (see the main text and "Methods").

**Figure 5:**
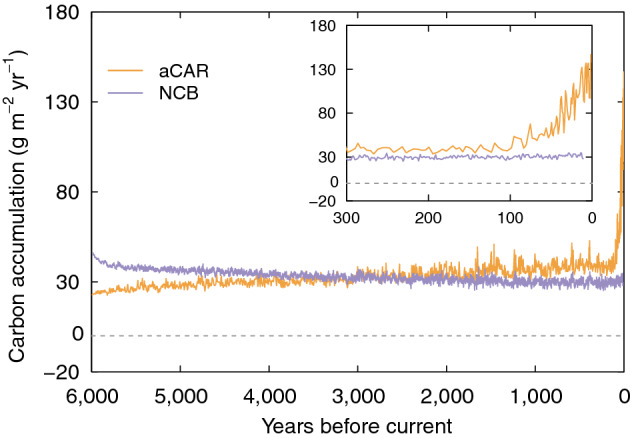
aCAR and NCB (20 year moving average) for the baseline simulation (see "Methods"). The aCAR values are for a virtual core taken from halfway down the modelled hillslope at the end of the simulation. NCB is calculated during each year of the simulation (i.e. at the time peat is gained or lost) and is akin to measuring C fluxes. The inset shows the typical ‘uptick’ of aCAR in recently-accumulated peat layers seen in many peat cores.

**Figure 6:**
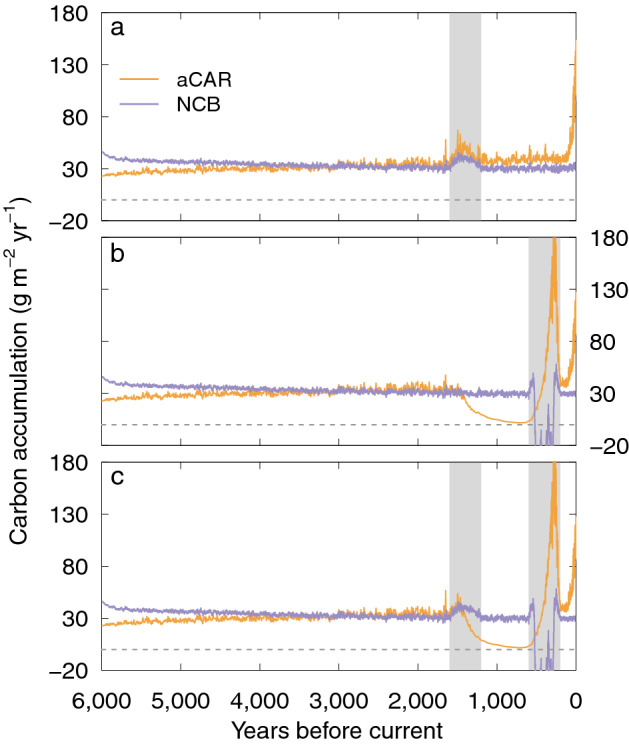
The effect of climate perturbations on simulated aCAR and NCB (20 year moving average). (**a**) Increase in temperature of 1.5 °C, (**b**) reduction in annual net rainfall of 0.4 m, and (**c**) both perturbations combined. The light grey vertical bars indicate the timing and duration of the perturbations and the dark grey dashed line is where C accumulation equals zero. The increases in NCB near to the beginning and end of the net rainfall perturbation (**a** and **b**) are due to the peatland water tables falling and later rising into the zone of maximum litter addition in DigiBog’s litter production equation.

Here we show aCAR and NCB from four simulations of the single blanket peatland (see "Methods" for details of the model set up). The results are shown in Figs. 4, 5 and 6. We used the net rainfall (precipitation minus evapotranspiration) and temperature inputs from^10^ for a baseline simulation (Figs. 4 and 5) and ran three modifications to the same dataset: (1) a 0.4 m reduction in annual net rainfall to simulate a long-term drought; (2) a 1.5 °C increase in air temperature to simulate a warming climate; and (3) the inputs from 1 and 2 combined (Fig. 6). All other input parameters remained unchanged from the baseline simulation (see "Methods"). To create the perturbations in driving data we linearly increased or decreased the input(s) over 100 years and allowed the simulation to continue using the modified data for a further 200 years before reversing the increase to use the original time series for the remainder of the model run (the total time of a modification—400 years—is henceforth known as the perturbation). We implemented the temperature perturbation (Fig. 6a) earlier than the one for net rainfall (Fig. 6b) so that we could see the effect of later events on aCAR and NCB (Fig. 6c) (see "Methods" for the details and timings of the driving data perturbations).

Our more detailed model shows aCAR and NCB (Fig. 5) conform to a pattern similar to the one given by our simple peatland model in Scenario 2 (Fig. 2). These dynamics are also predicted by the models used by^11,15^, and clearly show that aCAR is not the same as NCB. The modelled ‘uptick’ in aCAR in recently accumulated peat (towards the right-hand side of Fig. 5) is also seen in real cores taken from peatlands in a wide range of environments^10^. The uptick is due to the ‘acrotelm effect’ explained earlier.

The climate perturbations in Fig. 6 further illustrate why aCAR should not be used to represent NCB. Although they don’t have the same values as each other, aCAR and NCB in Fig. 6a increase and decrease similarly during the period in which the temperature perturbation occurs. This correspondence is because warming has shifted the peatland’s mass balance to be more in favour of plant litter production than the losses from decomposition. In this instance it might seem reasonable that aCAR can be used to indicate NCB. However, in Fig. 6b, the picture is more complicated and aCAR and NCB produce very different responses. The reduction in net rainfall deepens the peatland’s water tables, shifting the mass balance in favour of decomposition (i.e. all losses exceed all gains), but the changes in aCAR do not coincide with the timing of the perturbation. Whilst NCB is affected at the time of the climatic drying and becomes negative (there is an overall loss of C), the effects on aCAR are offset, but at no time is aCAR negative (it cannot be, as we explain earlier and as explained by^11^). aCAR suggests that C accumulation has reduced before the perturbation takes place but NCB has not actually changed at this time. This mismatch is because, as well as continuing to decompose, a peat layer can be altered by events that take place many years after it was originally formed. The reduction in aCAR shown in Fig. 6b is known as secondary decomposition or decay^9,33^. There follows a significant increase in aCAR ‘apparently’ indicating that C accumulation is also increasing when, in fact, the peatland is losing C as shown by NCB. This apparent increase in C is because a shift to deeper water tables can increase plant production; i.e., the mass added to the peatland increases. But because aCAR does not include the C fluxes from the whole peat column it does not take account of the increase in decomposition (the mass lost), and so the total change in C stored is not seen. Our simple peatland model in Fig. 3 also demonstrates how this difference between aCAR and NCB occurs.

Finally, when the perturbations are combined (Fig. 6c), the increase in aCAR around 1,600 years ago, caused by an increase in temperature, is partially wiped away by secondary decomposition before sharply declining, but NCB remains unchanged. Whilst it is likely that an assessment of aCAR would conclude that C accumulation had reduced during the time when the temperature was perturbed, the interpretation of both the timing and the magnitude of the reduction would be wrong. And the increase in aCAR starting around 600 years ago would also be misinterpreted as an increase in C accumulation rather than an overall reduction in the peatland’s C store.

### Implications for assessing changes in peatland C accumulation rates

Our simple peatland model, mass balance equations and DigiBog simulations, along with evidence from previous studies^10,11,14,15,17,19^ show that aCAR and RERCA cannot generally be used to assess changes to the rate of peatland C accumulation (NCB). Therefore, studies that use aCAR to indicate changes in peatland carbon balance processes over time (acrotelm effect) or to estimate NCB are unreliable and should be viewed with considerable circumspection.

As our simple peatland model scenarios show, in general, aCAR does not equal NCB^11^. Because all peatlands accumulate C according to the mass balance equation—i.e. assessing a peatland’s C balance requires that *all* of the peatland profile is taken into account and not just a dated section of it—our results apply to all peatlands in all circumstances. The only instance when we can be sure that aCAR equals NCB is when it is calculated for the whole of a peatland’s developmental history^11^. However, an average C accumulation rate for the entire history of the peatland is of limited use; land managers, researchers and policy makers are usually interested in how NCB has changed over time in response to climate and land-use. Although in some other circumstances (e.g. Fig. 6a) it appears that aCAR and NCB are sufficiently similar for aCAR to be useful (and sometimes they coincide—see Fig. 6 and^11^), this assessment can only be made because we can calculate NCB from our model outputs and compare the two quantities. But, unless NCB is known from C flux measurements or model simulations of peatland development, the correspondence of aCAR to NCB cannot be established.

The results of our simulations, and those from other studies^10,11^, also show how some land uses or changes to the climate may cause further mismatches in the timing, magnitude and sign of aCAR and NCB. Although acknowledging that aCAR gives an erroneous estimate of past rates of NCB, Frolking et al.^11^ do not advocate abandoning its use. Based on our evidence, and that provided by other studies, we suggest there is a need to go further. Given that aCAR is based on a mistaken use of the balance equation and can give the wrong sign of NCB as well as the opposite trend, we believe that it should no longer be acceptable to use aCAR to indicate changes in NCB.

Our simulations produce virtual and not real peat cores, and, by necessity, all models are simplifications of reality. The perturbations to our driving data are at the high end of what might be experienced naturally but are not implausible. They allow us to see more clearly how such events might affect the timing, magnitude and sign of aCAR in comparison to NCB. If our model is configured for a different type of peatland (e.g.^10^ simulated a raised bog) with different driving data or changes to land use, the results for aCAR and NCB will likely be different from the ones we show here. But despite these differences we would still not be able to reliably predict NCB from aCAR.

The challenge of understanding if C accumulation rates have been altered by external forcing has been discussed in the literature since^14^, but the implications of using aCAR to indicate NCB have recently been brought to the fore because of the imperative to assess the impact of climate change and land use on peatland C cycling. By highlighting and explaining the deficiencies of aCAR, our aim is to encourage the use of more robust and reliable approaches for calculating past actual C accumulation rates (NCB). Ideally, direct measurements of C fluxes would be used^10^, but such observations are not available for many sites and, where they do exist, they will cover only the last few decades at most (see above). Therefore, for C accumulation histories extending to centuries and millennia, we propose that C balance models fitted to peatland age-depth (or age-mass) curves are used to estimate if NCB has changed over time. Simple models—for example, that of^14^—are already used in this regard and are worthy of further investigation^19,28,34–36^. For example, several studies have derived peatland NCB at the global^28,37^ regional^35^, and local^17,19^ scales. The authors back-calculate NCB from the net C pool using empirical models that consider autogenic long-term peat decomposition^14^. In a further step, with the aim of understanding if contemporary C accumulation rates were different from past rates^17^ and^19^ compared the calculated NCB from the catotelm to predictions of peat C mass transfer at the acrotelm-catotelm boundary, using a forward model of acrotelm peat decay. That being said, these approaches cannot differentiate the effects of long-term autogenic decay on peat versus that of secondary decomposition, which could be brought about by land-use or climate change.

Given the limitations of such approaches, we encourage exploration of the potential of fitting more complete ecosystem models like the Holocene Peat Model^38^, MILLENNIA^39^ and DigiBog to data from peat cores to help estimate changes in peatland function over time. Observations of peat depth and downcore humification along with the inclusion of proxy data from the peatland in question—often shown in palaeoecological studies—are also important for contextualising model outputs^10^.

In conclusion, aCAR is an unsuitable proxy for the actual C accumulation rates of peatlands. Approaches that conceptualise peatlands as dynamic C stores—the balance of all mass additions and losses - are needed. And, as we have noted, some studies, recognising the problems of aCAR, have provided potential alternatives. However, to be useful, it is likely that existing models will need to be modified, tested and their suitability assessed, or new ones developed so that credible comparisons of the effect of climate change or land uses on peatland C accumulation rates can be made.

## Methods

### Model set up and driving data

In^10^ we simulated a 2-D transect of raised bog with a radius of 150 m (75 × 2 m × 2 m columns). Here we used the same length transect over a sloping impermeable mineral soil base (3°). A no-flow condition at the top of the slope represented a drainage divide, and a fixed water level condition at the downslope margin represented a stream. The model was set up as described by^40^ with the parameter values shown in Table 1. The parameters fall within the range of values used for previous DigiBog simulations^33,40^. We used the version of DigiBog described by^10^. This version of the model is spatially explicit, comprising hydrologically connected columns that, depending on its configuration, form a 2-D or 3-D peatland. DigiBog simulates the accumulation of peat over millennial timescales by adding layers of plant litter to each column (the thickness of which depends on mean annual air temperature and water-table position) and by decomposing existing peat layers. Decomposition of whole or part peat layers is also dependent on air temperature and on water-table position (peat above the water table is exposed to a greater rate of decay according to the parameters in Table 1). Water-table position is determined in a hydrological sub-model that simulates water movement between columns depending on peat hydraulic conductivity (which the model calculates based on the decomposition of a layer), water-table gradient, and the model’s driving data (precipitation minus evapotranspiration). Detailed descriptions of DigiBog’s routines can be found in^40^.

**Table 1:** Model parameter set.

Parameter	Symbol	Value	Units
Oxic decomposition	*α*_*ox*_	3.5 × 10^–2^	year^−1^
Anoxic decomposition	*α*_*an*_	10^–4^	year^−1^
Temperature sensitivity	*Q*_10_	3	–
Dry bulk density	*ρ*	100	kg m^−3^
Hydraulic conductivity parameter	*a*	15.8687	m year^−1^
Hydraulic conductivity parameter	*b*	8	–
Drainable porosity	*s*	0.3	–
Peat column size	–	4	m^2^
Pond depth	–	2.5 × 10^–3^	m

**Figure 7:**
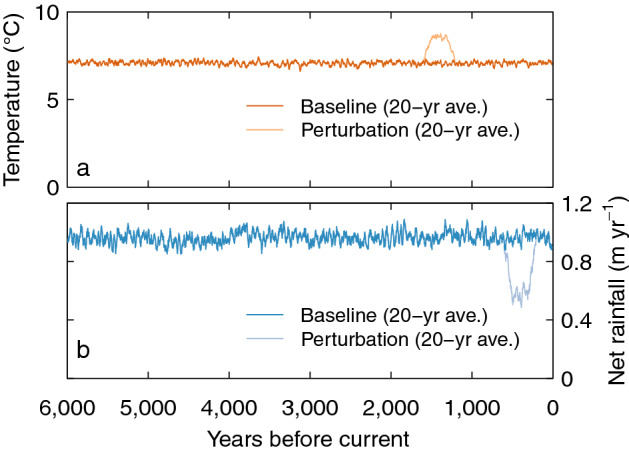
Modifications to the driving data for the simulations shown in Fig. 6. (**a**) Temperature and (**b**) net rainfall. The perturbations show the timing and duration of the deviations from the baseline simulation inputs.

Our baseline simulation used the driving data from^10^. We modified these data with perturbations to temperature and net rainfall of 400 years duration starting 1600 years and 600 years ago respectively (as described in the main text and shown in Fig. 7).

### Calculating aCAR and NCB from model outputs

In DigiBog, new peat layers are added to each column at the end of every year of a simulation. Throughout a simulation, the model keeps track of the age and the decreasing mass (g m^−2^) of a layer which enabled us to calculate aCAR. We selected one of the columns from the middle our simulated peatland (column 37—i.e. its centre was 77 m from the drainage divide) and, starting at the base of this virtual core, we calculated the average mass of each pair of layers (i.e. layers 1 and 2, layers 2 and 3, etc.) and divided the result by the age difference in years between the two layers. To estimate the rate of C accumulation, the resulting peat mass was multiplied by 0.5^17^. The model also records the height of each peat column at the end of each year. To calculate the change in C stock (NCB) between two consecutive years, we subtracted the height of a peat column at year *t*_*i*-1_ from that at year *t*_*i*_ and converted the change in height into a change in peat mass (our peat has a bulk density of 100 kg m^−3^)—that is to say, we followed Eq. (3), ${\Delta S}_{p}/\Delta t=\left({S}_{p,i}-{S}_{p, i-1}\right)/\left({t}_{i}-{t}_{i-1}\right)$—and again multiplied the result by 0.5 to give the change in the C store. We compared the mean annual NCB with the Long-Term Rate of C Accumulation (LORCA)^11^ (Table 2). LORCA (M L^−2^ T^−1^) is calculated for the whole of a peatland’s developmental history (a dated layer of 6000 years in our case) and therefore accounts for all the additions and losses of C during this time (i.e., it is the change in the mass of $\mathrm{C}= {\Delta S}_{p}/\Delta t$). As a result, mean annual NCB and LORCA should be the same^11^ and they were (Table 2).

**Table 2:** Comparison of LORCA and NCB (g C m^−2^ year^−1^) for the DigiBog simulations shown in Figs. 5 and 6.

Simulation	LORCA	Mean NCB
Baseline (Fig. 5)	33.18	33.18
Temperature perturbation (Fig. 6a)	33.72	33.72
Net rainfall perturbation (Fig. 6b)	29.89	29.89
Combined perturbation (Fig. 6c)	30.16	30.16

## Data Availability

DigiBog model outputs are available from Dylan M. Young on reasonable request.
